# King post truss as a motif for internal structure of (meta)material with controlled elastic properties

**DOI:** 10.1098/rsos.171153

**Published:** 2017-10-18

**Authors:** Emilio Turco, Ivan Giorgio, Anil Misra, Francesco dell’Isola

**Affiliations:** 1 DADU, University of Sassari, Sassari, Sassari, Italy; 2 DISG, University of Roma ‘La Sapienza’, Rome, Italy; 3 CEAE, The University of Kansas, Lawrence, KS, USA

**Keywords:** metamaterial design, nonlinear behaviour, king post truss

## Abstract

One of the most interesting challenges in the modern theory of materials consists in the determination of those microstructures which produce, at the macro-level, a class of metamaterials whose elastic range is many orders of magnitude wider than the one exhibited by ‘standard’ materials. In dell’Isola *et al.* (2015 *Zeitschrift für angewandte Mathematik und Physik* **66**, 3473–3498. (doi:10.1007/s00033-015-0556-4)), it was proved that, with a pantographic microstructure constituted by ‘long’ micro-beams it is possible to obtain metamaterials whose elastic range spans up to an elongation exceeding 30%. In this paper, we demonstrate that the same behaviour can be obtained by means of an internal microstructure based on a king post motif. This solution shows many advantages: it involves only microbeams; all constituting beams are undergoing only extension or compression; all internal constraints are terminal pivots. While the elastic deformation energy can be determined as easily as in the case of long-beam microstructure, the proposed design seems to have obvious remarkable advantages: it seems to be more damage resistant and therefore to be able to have a wider elastic range; it can be realized with the same three-dimensional printing technology; it seems to be less subject to compression buckling. The analysis which we present here includes: (i) the determination of Hencky-type discrete models for king post trusses, (ii) the application of an effective integration scheme to a class of relevant deformation tests for the proposed metamaterial and (iii) the numerical determination of an equivalent second gradient continuum model. The numerical tools which we have developed and which are presented here can be readily used to develop an extensive measurement campaign for the proposed metamaterial.

## 1. Introduction

Recent advances in computer-aided material and structural fabrication, such as rapid prototyping, three-dimensional (3D) printing or additive manufacturing, have created avenues for designing materials with tailored behaviour. These technological developments have also opened unexpected fields of applications of ancient mathematical problems and methods, whose applications were originally found in a different context. Indeed, the revolutionary and somehow underestimated contribution by Maxwell [1] allowed for the exact design, already in 1864, of a wide class of beam lattices; an approach used later (owing to the vulgarization due to Mohr [2]) for building bridges and, in general, many kinds of civil engineering structures. This approach due to its mathematical generality, can be applied to design also lattice microstructures constituting novel modern metamaterials. Maxwell efficiently applied the principle of virtual work to calculate the relevant physical quantities in truss structures using efficient algorithms: in the absence of automatic computation tools Maxwell methods were exploited efficiently by means of Cremona diagrams (e.g. [3]), which transformed the mathematical visions by Maxwell into engineering artefacts (for a careful historical discussion of some of this subject, see [4]).

While it is nowadays not useful in practice to use Cremona diagrams (notwithstanding the fact that they are still taught in many engineering curricula), the basic mathematical ideas by Maxwell represent, till now, the main conceptual tool for investigating the mechanical behaviour of any lattice of beams (also those which are not trusses!). In the present paper, we used the principle of virtual work, exactly in the Maxwellian spirit, but we used modern calculation tools: i.e. von Neumann machines and effective algorithms for finding minima of deformation energy. Energetic methods are useful not only for finding the equilibrium configuration of beam lattices, but also for determining their optimal topological and mechanical configurations targeted to specific functional objectives.

In more recent times, using a similar top-down approach, topology optimization is being exploited for designing materials and structures that can be manufactured using additive manufacturing [5,6] as well as to develop material microstructure for concurrent multi-scale design of composite macrostructure [7], or exploit peculiar micro-scale properties to propose optimal structural topologies [8]. Furthermore, metamaterials with exceptional elastic properties have been fabricated using 3D printing techniques [9,10]. Most of these material designs are conceived within the framework of classical continuum mechanics and yield materials that conform to the properties predicted by the classical theories. Alternatively, from a bottom-up approach microstructure can be designed by carefully selecting the unit building block to obtain ‘metamaterials’ of specified properties, such as granular metamaterials with frequency band gaps or specific architectures that have a significant effect of microstructure on macro-scale behaviour [11,12]. Internal structures that replicate certain macro-scale truss systems could yield unique material-scale behaviour (for some interesting applications, see [13–16]).

In the present work, we explore the development of metamaterials by using a king post truss as the underlying motif. Through careful simulations, we show that by combining the king post truss into pantographic microstructures, it is possible to obtain metamaterials with unique and controlled strain energy responses. In the subsequent discussion, we first present a brief description of the simulation methodology, we then describe the response of the unit building block, and subsequently, we simulate the behaviour of two-dimensional structure under tension, shear and bending. The simulation results show that the proposed metamaterial has unique elastic properties that includes phase transition-like behaviour. From the viewpoint of macro-scale modelling the proposed metamaterial exhibits strong second gradient continuum behaviour, such as the predicted zones of quasi-uniform deformation with transition zones (boundary layers) of finite thickness.

## 2. Pantographic lattice with king post truss building block

### 2.1. Mathematical formulation

**Figure 1.**
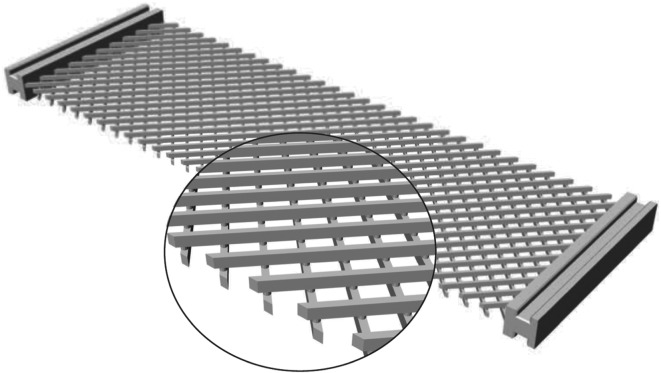
Rendering of a pantographic lattice specimen.

A class of planar metamaterials has been recently extensively studied (called pantographic sheets in [17] and studied in detail in [18–21]) showing a rather exotic mechanical behaviour. Their microstructure can be recognized in figure 1, in which a specimen obtained by using 3D printing technology is shown.

We can distinguish two families of fibres, which behave as beams, connected to each other by means of cylindrical connections named pivots. Various papers, see for example [17,22,23], show, from a theoretically point of view, through numerical simulation and also by experiments, that this kind of metamaterial behaves elastically under very large deformations; see for example [24] for the case of load applied on single fibres, [25,26] which discuss some non-standard tests such as traction and bending and [27] for a review on pantographic fabrics; see also [28,29] for a general theoretical introduction on discrete system, [30] for non-local structural elements and [31] for fracture phenomena.

**Figure 2.**
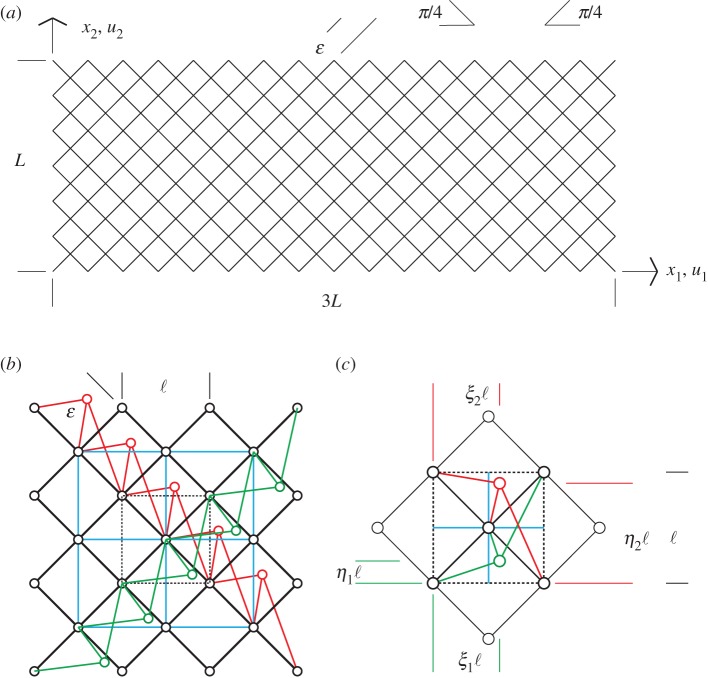
King post pantographic lattice: geometry (*a*), pantographic rods (in black), king post rods (in red and green), auxiliary rods (in cyan) (*b*) and king post geometric parameters (*c*).

This peculiar elastic behaviour, which has many potential practical applications, served as a motivation to design the building block unit of this lattice as that illustrated in figure 2 making use of the king post truss scheme; see [32,33].

The success of the design efforts obtained up to now justified a further conceptual development, which seems to us to deserve attention. Indeed, in the present paper we show that it is possible to conceive a microstructure constituted by a system of micro-trusses (i.e. systems of beams in which each constituent beam can be deformed only in compression or in extension) whose macro-scale behaviour is very similar to the pantographic sheets investigated up to now. This solution shows some relevant advantages, as it is well known that beams are extremely resistant to traction and, also when undergoing buckling, they are efficient when subject to compression. Moreover, we are confident that the truss microstructure which we designed can be realized using available 3D printing technology.

In our scheme, we distinguish the pantographic beams (depicted in black; see figure 2*a*–*c*), the king post rods (in red and green) which ensure the bending stiffness for each beam, and the auxiliary rods (in cyan), which prevent lattice rigid body motions. The geometry of the king post rods is completely defined by using two couples of geometric parameters such as reported in figure 2*c*, i.e. (*ξ*_1_,*η*_1_) and (*ξ*_2_,*η*_2_). Further, in this paper, we consider two-dimensional sheet structures, as that shown in figure 1, that can be potentially combined to form laminates or other 3D structures. With a view to focusing our discussion to the in-plane behaviour of these sheet structures, we remark that no out-of-plane displacements are considered. We also note that the simulation results presented in the subsequent discussions are for the case of orthogonal fibres; however, the extension to the more general case of non-orthogonal fibres is possible as described in [34].

The king post pantographic lattice naturally considers a set of Lagrangian parameters specifying the position of nodes, both those formed at the connections of the pantographic bars as well as those for king post bars. These parameters are initially located at the nodes of the reference configuration. Upon the in-plane deformation of the sheet, the nodes and the corresponding parameters displace to the current configuration. For planar motions, only a set of 2*n* coordinates is sufficient (if *n* is the number of considered nodes) the generic designation for which in the reference position is given by *P*_*i*_. In the deformed configuration, the set of Lagrangian coordinates of the corresponding position are denoted by *p*_*i*_.

The strain energy of the discrete model is the only kind of energy to be specified in the absence of relevant volume forces. The postulated expression for the Lagrangian discrete deformation energy *W*_int_ (in terms of the Lagrangian coordinates *p*_*i*_) is completely defined, specifying the contribution of the *e*th bar between the *i*_*e*_ and *j*_*e*_ nodes:

2.1 $$W_{e}=\frac{1}{2}a_{e}{\left(∥p_{j_{e}}-p_{i_{e}}∥-∥P_{j_{e}}-P_{i_{e}}∥\right)}^{2},$$

where *a*_*e*_ is the axial stiffnesses of the *e*th bar.

To have a complete solution of the considered equilibrium problem, i.e. the displacements (from which can be easily evaluated the structural reaction), a step-by-step procedure was implemented to reconstruct the complete equilibrium path of the king post pantographic sheet.

The total energy of the pantographic structure can be computed in a straightforward manner by simply adding the strain contribution of each bar. Formally, we can write

2.2 $$W(\text{d})=W_{\mathrm{int}}-W_{\mathrm{ext}}=\sum_{e=1}^{n_{e}}W_{e}-W_{\mathrm{ext}},$$

where *e* ranges on all the *n*_*e*_ bars, *W*_ext_ is the work of the external loads and all quantities on the right-hand side depend on the vector **d**, which collects the nodal displacements of the king post pantographic lattice.

As the equilibrium problem which we want to consider is a mixed one, we assume that the displacements of some nodes are imposed and that some externally conservative forces are applied to the remaining nodes. Let us therefore decompose **d** into the pair composed by two vectors: the assigned displacements **u**_*a*_ and the free displacements **u**. For notational aims, we will reorder **d** to get the decomposition

$$\text{d}=(\text{u},{\text{u}}_{a})$$

and, based on our assumptions, *W*_ext_ depends only on **u**.

The nonlinear system of equilibrium equations is obtained by imposing that the first variation of *W* vanishes, according to the following formula:

2.3 s(u)−p(u)=0,

where **p**(**u**) is the vector which collects the Lagrangian components of external forces (which may be assumed to be dead loads, for instance, so that **p** becomes independent of **u** and **s**(**u**) is the vector of the internal forces (also called, in the context of structural mechanics, *structural reaction*), as defined by

2.4 $$\text{s}(\text{u})=\frac{dW_{\mathrm{int}}}{d\text{u}}\;\mathrm{and}\;\text{p}(\text{u})=\frac{dW_{\mathrm{ext}}}{d\text{u}}.$$

The tangent stiffness matrix is defined as the derivative of the structural reaction **s**(**u**) with respect to the displacement vector **u**, according to the following formulae:

2.5 $${\text{K}}_{T}(\text{u})=\frac{d\text{s}(\text{u})}{d\text{u}}=\frac{d^{2}W_{\mathrm{int}}}{d{\text{u}}^{2}}.$$

The solution of the nonlinear equilibrium system of equations (2.3) can be found by means of an incremental–iterative procedure essentially based on the Newton scheme. As we will limit ourselves to the case of equilibrium paths depending only on imposed displacements, we introduce here only the parameter **λ**. Starting from an estimated point of the equilibrium path (λ_*j*_,**u**_*j*_) verifying that the residue **r** of equation (2.3) is

2.6 $$∥\text{r}({\text{u}}_{j},\lambda_{j})∥\leq \eta ,$$

i.e. with a pair being an *η*-approximate solution of the equilibrium condition (2.3), the iterative scheme, once the step Δ*λ* is fixed, is obtained by constructing the *η*-approximate solution (**u**_*j*+1_=:**u**_*j*_+Δ**u**_*j*_, **λ**_*j*+1_:=**λ**_*j*_+Δλ) by using the iterative scheme based on the Riks’ arc-length parameter to closely follow the equilibrium path. This effective algorithm is well described in [23] for a nonlinear elastic problem and in [35] for an elasto-plastic problem with some insights into filtering properties of such a procedure.

### 2.2. Behaviour of king post truss

**Figure 3.**
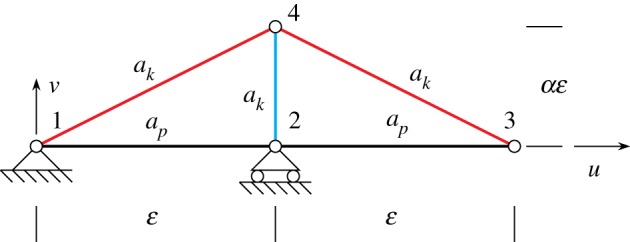
King post truss: geometry and rod stiffnesses.

Our exploration of the mechanical behaviour of the king post pantographic lattice begins by studying the response of the basic structural scheme sketched in figure 3. The five rods of the scheme connecting the four nodes can be distinguished in pantographic rods (in black) and king post rods (in red and cyan)^1^ (see also figure 2*b*,*c*), and fixes the attention, e.g. on the fibres which embrace the red king post.

We studied the strain energy *W* of the structure corresponding to the displacement triplet *u*_2_, *u*_3_ and *v*_3_ which have to be considered as imposed displacement. Nonlinear elastic solution of this problem was obtained by using the tool illustrated in the foregoing §2.1. The strain energy is evaluated using the following cases: geometry, *ε*=1, *α*=0.5; stiffnesses: *a*_p_=1, *a*_k_=0.01, 1, 100; range of displacements: −0.3≤*u*_2_≤0.3, −0.6≤*u*_3_≤0.6 and −0.6≤*v*_3_≤0.6; representing 30% deformation from the original configuration in the *u*-direction and 120% in the *v*-direction.

To examine the behaviour of the king post truss under different deformation conditions, we define the following strain parameters *δ*_12_, *δ*_23_ and *β*:

2.7 $$\delta_{12}=u_{2},\;\delta_{23}=\sqrt{{\left(\varepsilon +u_{3}-u_{2}\right)}^{2}+v_{3}^{2}}-\varepsilon \;\mathrm{and}\;\beta =\arctan\frac{v_{3}}{\varepsilon +u_{3}-u_{2}},$$

where *δ*_12_ is the elongation (compression) of the pantographic rod 1–2, *δ*_23_ is the elongation (compression) of the pantographic rod 2–3 and *β* is the angular distortion of the truss.

**Figure 4.**
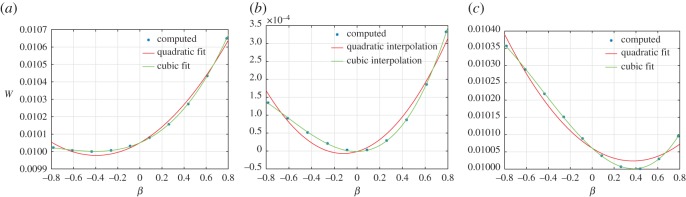
King post truss: strain energy *W* varying the angular distortion *β* for the cases *δ*_12_=*δ*_23_=−0.1 (*a*), 0 (*b*) and 0.1 (*c*).

As examples of the king post truss behaviour, we plot the strain-energy variation in figure 4 for particular cases of the above strain parameters and the stiffness parameter ratio *a*=*a*_k_/*a*_p_=0.01. For example, in figure 4 the strain energy *W* is plotted by varying the angle *β*, in the range (−*π*/4,*π*/4), while holding the elongations *δ*_12_=*δ*_23_=−0.1 (*a*), 0 (*b*) and 0.1 (*c*). We also give the best fit to the strain-energy variation using both quadratic and cubic fitting. We remark that the king post truss does not have a symmetrical solution in accordance with the lack of symmetry of the geometry.

**Figure 5.**
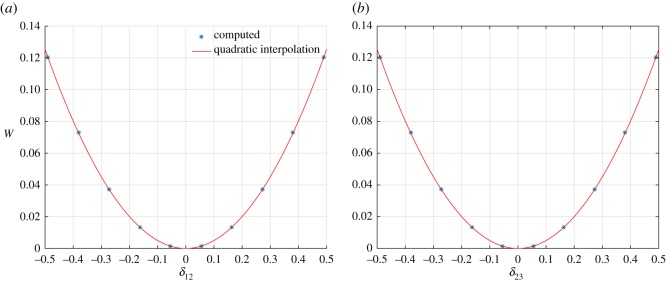
King post truss: strain energy *W* varying *δ*_23_ (*a*) (*β*=*δ*_12_=0) and *δ*_12_ (*b*) (*β*=*δ*_23_=0).

Further in figure 5*a*, we plot *W*(*δ*_12_) for the case *δ*_23_=*β*=0 (−0.5≤*δ*_12_≤0.5) and in figure 5*b*, we plot *W*(*δ*_23_) (−0.5≤*δ*_23_≤0.5) while holding *δ*_12_=*β*=0, along with their respective best fit using quadratic fitting. We observe from these plots that, for the two elongation deformation modes, the strain energy of this basic system shows a quadratic variation, while for the angular distortion deformation mode the strain-energy variation is non-quadratic. The clear indication is that for mixed deformation modes the strain-energy landscape of the basic system can be expected to be complex and not follow a simple quadratic variation.

**Figure 6.**
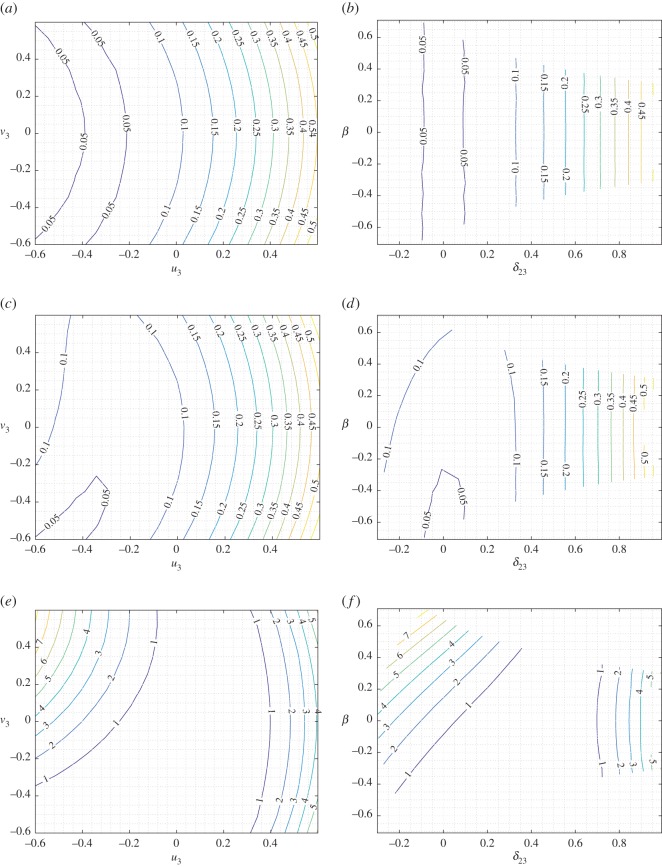
King post truss: level sets for the strain energy *W* for *δ*_12_=−0.3 varying *a* in the plane *u*_3_–*v*_3_ (*a*,*c*,*e*) and in the plane *δ*_23_–*β* (*b*,*d*,*f*). (*a*,*b*) *a*=0.01, (*c*,*d*) *a*=1 and (*e*,*f*) *a*=100.

**Figure 7.**
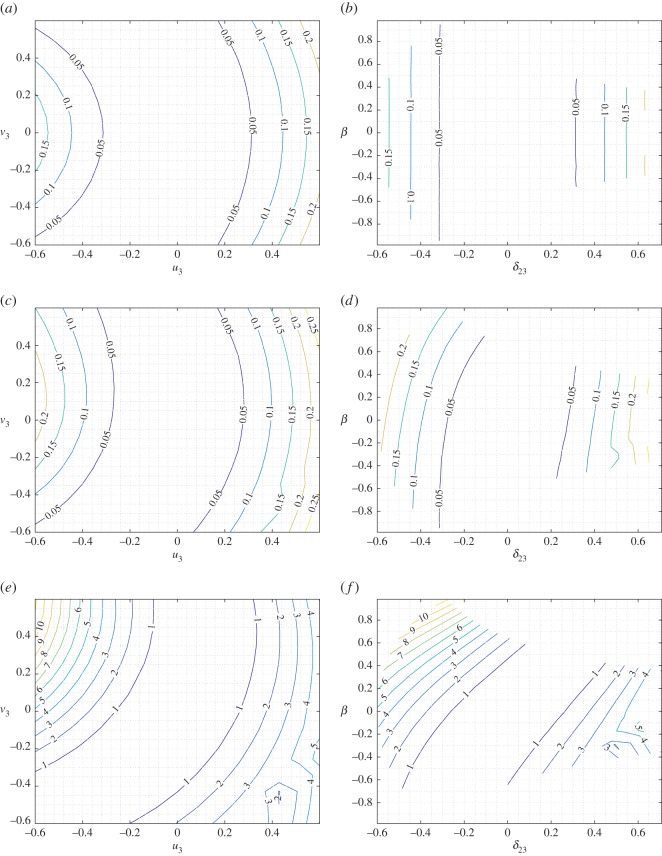
King post truss: level sets for the strain energy *W* for *δ*_12_=0 varying *a* in the plane *u*_3_–*v*_3_ (*a*,*c*,*e*) and in the plane *δ*_23_–*β* (*b*,*d*,*f*). (*a*,*b*) *a*=0.01, (*c*,*d*) *a*=1 and (*e*,*f*) *a*=100.

**Figure 8.**
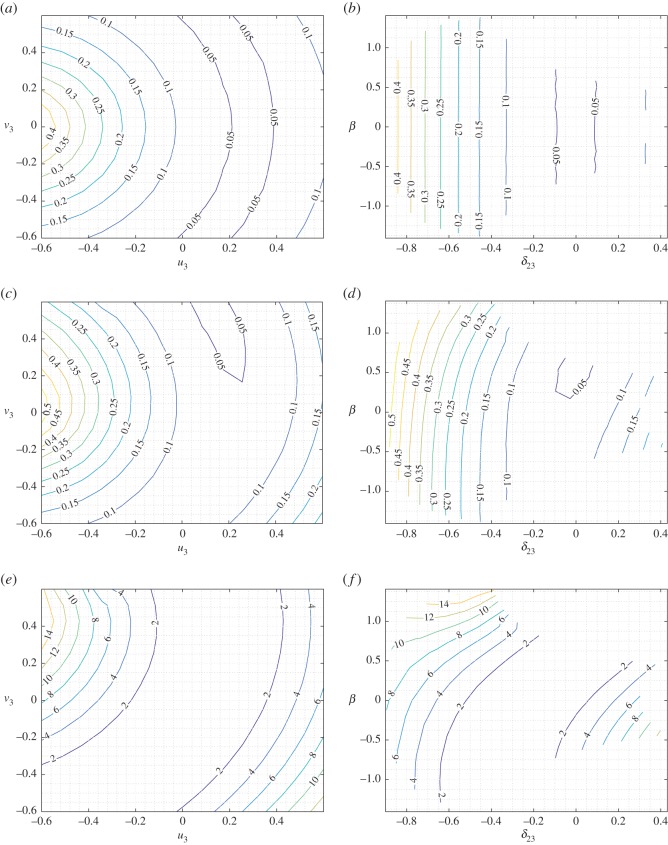
King post truss: level sets for the strain energy *W* for *δ*_12_=0.3 varying *a* in the plane *u*_3_–*v*_3_ (*a*,*c*,*e*) and in the plane *δ*_23_–*β* (*b*,*d*,*f*). (*a*,*b*) *a*=0.01, (*c*,*d*) *a*=1 and (*e*,*f*) *a*=100.

To obtain a better picture of the strain-energy variation under mixed deformations, we report in figures 6–8 selected level sets for the strain energy *W* for the three cases *u*_2_=−0.3,0,0.3, respectively, representing the shortening–elongation cases for pantographic rod 1–2 while the other two parameters *u*_3_ and *v*_3_ are varied in the range noted previously. In each of these figures, we give the level sets of the strain energy in the plane *u*_3_–*v*_3_ (panels *a*,*c*,*e*) and *β*–*δ*_23_ (panels *b*,*d*,*f*) for three different values of the stiffness parameter ratio as noted above. It is clear from the strain-energy level sets that a rich range of behaviour is exhibited by the basic system under mixed deformation. In material systems comprising a large number of these basic structures, the overall response will be some combination of these local responses. Comparison of figures 6 and 8 points to the asymmetry in the strain-energy behaviour induced by compression–tension (shortening–elongation) of the pantographic rod 1–2.

### 2.3. Behaviour of king post pantographic unit

**Figure 9.**
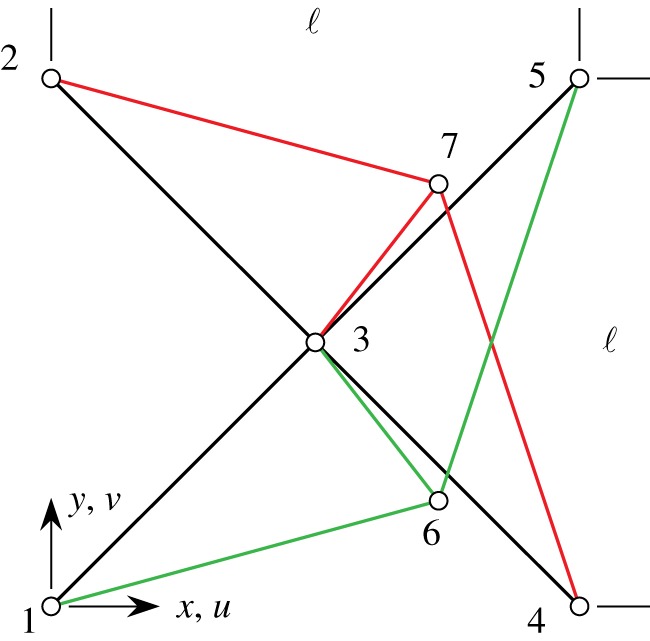
King post pantographic unit (the position of nodes 6 and 7 is slightly modified for the sake of illustration).

By incorporating the king post truss basic structure into a pantographic microstructure, it is possible to exploit its wide range of strain-energy response at the material scale. In a pantographic microstructure, the unit building block is represented as shown in figure 2*c* and isolated in figure 9. We analyse the response of this unit as a precursor indicator of the overall response of the envisaged metamaterial. The geometry of this unit, described by the nodal coordinates labelled from 1 to 7, are reported in table 1 (we stress that in figure 9 and in table 1 nodes are numbered in a completely independent way with respect to the nodes of figure 3). From them, it is easy to evaluate the reference length of each rod which depends only on the side ℓ of the square unit.

**Table 1.** Nodal coordinates *x*,*y* of the king post pantographic unit.

node	1	2	3	4	5	6	7
*x*	0	0	0.5ℓ	ℓ	ℓ	0.75ℓ	0.75ℓ
*y*	0	ℓ	0.5ℓ	0	ℓ	0.25ℓ	0.75ℓ

From these data, we perform a series of analyses changing the ratio *a*=*a*_k_/*a*_p_ between the stiffness *a*_p_ of pantographic rods and those of king post rods *a*_k_ (we assume that all the rods of the king post have the same stiffness parameter). We consider the following case study: nodes 1 and 2 are locked, whereas the same horizontal displacement *u* is assigned to the nodes 4 and 5, from zero until its maximum value $u_{\max}=0.55\ell$.

**Figure 10.**
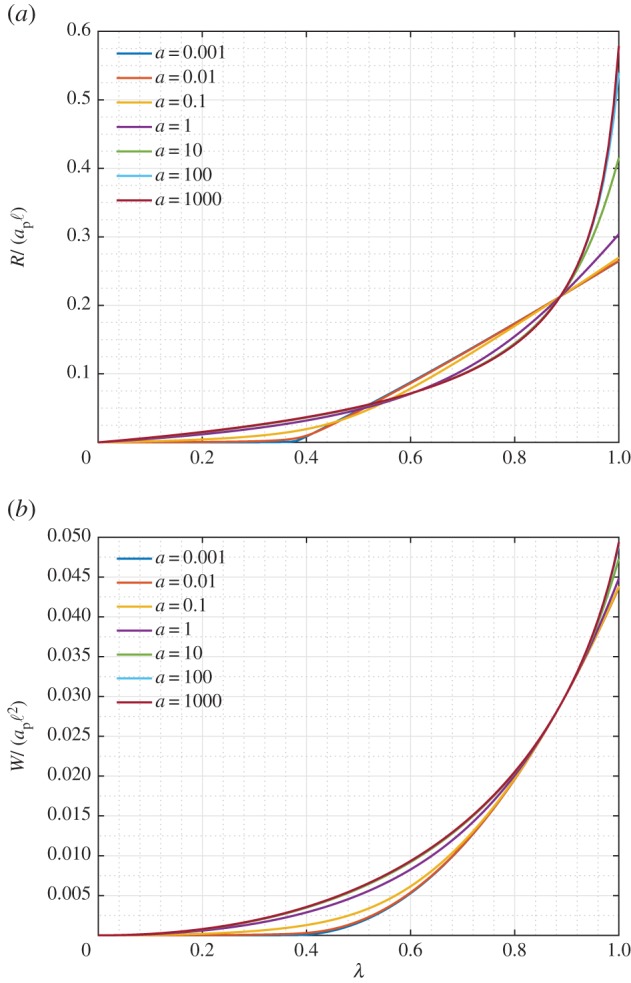
King post pantographic unit: evolution of the normalized structural reaction *R*/(*a*_p_ℓ) (*a*) and normalized strain energy *W*/(*a*_p_ℓ^2^) (*b*) versus the non-dimensional displacement parameter λ varying the ratio *a*=*a*_k_/*a*_p_.

Figure 10 reports, respectively, the normalized structural reaction *R*/(*a*_p_ℓ) (figure 10*a*) and the normalized global strain energy *W*/(*a*_p_ℓ^2^) (figure 10*b*) when the non-dimensional displacement parameter $\lambda =u/u_{\max}$ increases. A range of strain-energy response for the pantographic unit is obtained even under this relatively simple deformation mode. For the cases of ratio *a*<1, an abrupt change in the response is observed at certain elongation (λ≈0.39–0.4). This abrupt change is related to the angular distortion of the king post truss becoming significant beyond a certain elongation of the pantographic unit. It is noteworthy that the response of the pantographic unit has further enrichment in contrast to that of the king post truss, which can be further contrasted with the simple quadratic strain-energy behaviour of the rods that comprise the unit. The enriched behaviour is a clear consequence of the competition/collaboration of the deformation modes in this hierarchical structure.

**Figure 11.**
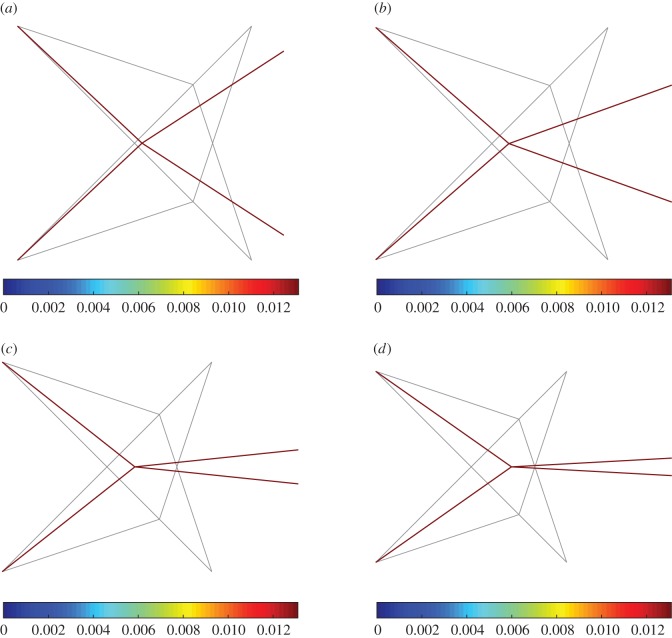
King post pantographic unit: deformation as λ increases for *a*=1 (the colour bar represents the global strain energy, the reference position is indicated in grey). (*a*) λ=0.25, (*b*) λ=0.5, (*c*) λ=0.75 and (*d*) λ=1.

Figure 11 reports the deformation for the case *a*=1 for λ=0.25,0.5,0.75,1. The colour bar represents the global strain energy. The reference configuration of the unit is shown in grey, while the deformed configuration is represented by the pantographic rods only (the king post rod has been removed for clarity).

**Figure 12.**
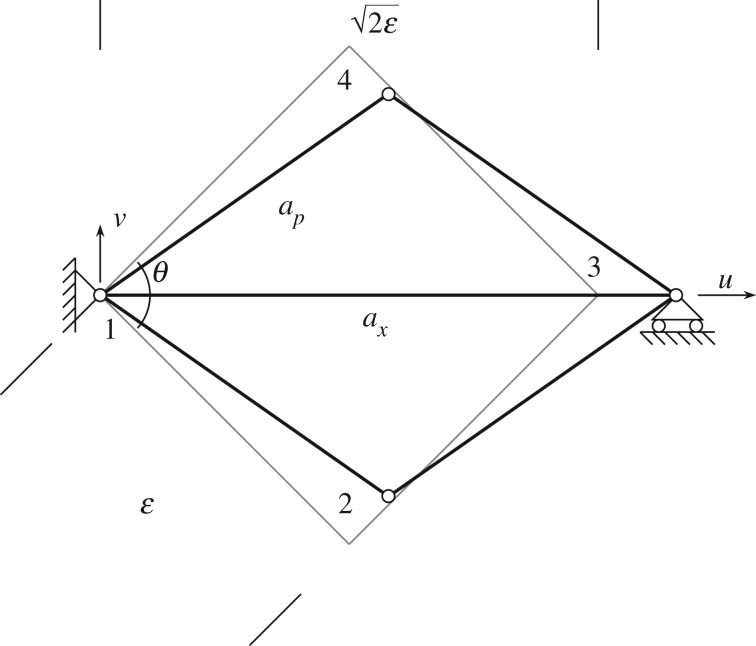
Geometric relationship between the strain of auxiliary rod and the shear deformation of a square field of the pantographic lattice.

It remains to clarify the quantitative contribution of the auxiliary rods. If we consider the simple structural scheme sketched in figure 12, it is simple to prove that, for a pantographic lattice with orthogonal fibres,

2.8 $$u_{3}=\left(2\cos\frac{\theta }{2}-\sqrt{2}\right)\varepsilon ,$$

which reveals the auxiliary rods’ contribution to contrast the shear mode of the square formed by the four pantographic rods (figure 2*b*). This relationship could be useful to calibrate the stiffness of the auxiliary rods, following, for example, the guidelines reported in [36–38].

## 3. Numerical simulation under extension, shear and bending

We now consider the numerical simulation of the pantographic lattice sheet subjected to standard mechanical tests, including extension, shear and bending. Using the foregoing experience gained from the king post truss and the king post pantographic unit, and taking into account the relationship (2.8), we assume the following data (figure 2):

(i) geometry: *L*=66.114; $L/\varepsilon =4\sqrt{2}$; *ξ*_1_=0.75; *η*_1_=0.25; *ξ*_2_=0.75; *η*_2_=0.75;
(ii) stiffnesses: *a*_p_=1; *a*_*x*_/*a*_p_=10^−5^.

The assumed displacements for the three numerical simulations are

extension test *u*_1_(0,*x*_2_)=*u*_2_(0,*x*_2_)=*u*_2_(3*L*,*x*_2_)=0 and $u_{1}(3L,x_{2})=u_{1}^{\max}=75$;
  shear test *u*_1_(0,*x*_2_)=*u*_2_(0,*x*_2_)=*u*_1_(3*L*,*x*_2_)=0 and $u_{2}(3L,x_{2})=u_{2}^{\max}=75$;
bending test *u*_1_(0,*x*_2_)=*u*_2_(0,*x*_2_)=0 and $u_{2}(3L,x_{2})=u_{1}^{\max}(1-2(x_{2}/L))$, $u_{1}^{\max}=35$.

**Figure 13.**
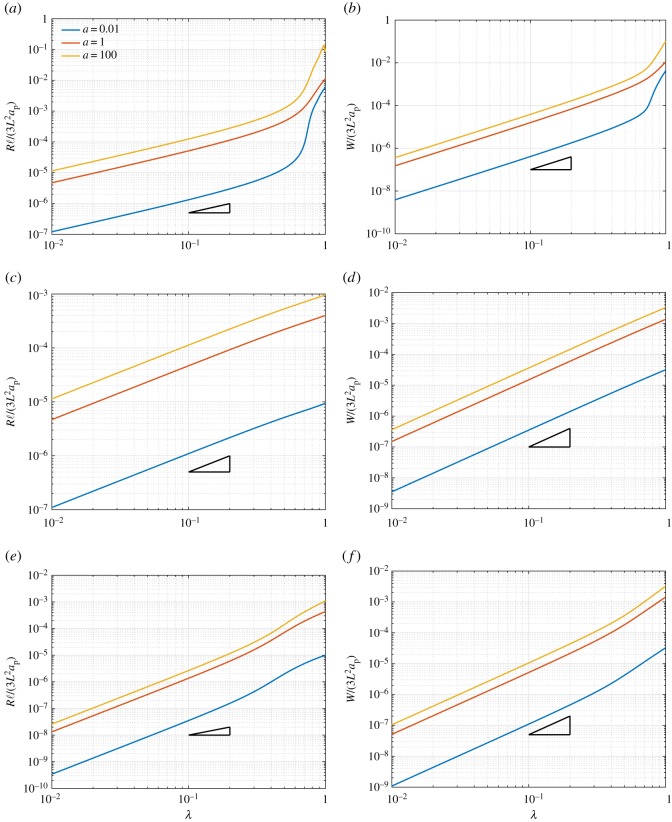
Extension, shear and bending tests: non-dimensional structural reaction in the *x*-direction *R*ℓ/(3*L*^2^*a*_p_) (*a*,*e*) and the *y*-direction *R*ℓ/(3*L*^2^*a*_p_) (*c*) and non-dimensional global strain energy *W*/(3*L*^2^*a*_p_) evolution (*b*,*d*,*f*) varying *a*=*a*_k_/*a*_p_ and taking $u_{1}^{\max}=75$ for the extension test (*a*,*b*), $u_{2}^{\max}=75$ for the shear test (*c*,*d*) and $u_{1}^{\max}=35$ for the bending test (the black triangle indicates the slope of the linear law for the structural reaction and the quadratic law for the strain energy).

Figure 13 reports the non-dimensional structural reaction *R*ℓ/(3*L*^2^*a*_p_) (figure 13*a*,*c*,*e*) and the non-dimensional global strain energy *W*/(3*L*^2^*a*_p_) (figure 13*b*,*d*,*f*) for the extension, shear and bending cases, respectively. In these plots, we indicate using a black triangle, the slope of the linear law (λ) for the reactions and the quadratic law (λ^2^) for the strain energies. These simulations amply demonstrate that the proposed material system exhibits non-classical elastic behaviour even though its fundamental components are classical rods. Particularly notable is the completely reversible phase transition from a relatively soft and linear to stiff and hardening material at critical extension and bending, while retaining a classical behaviour in shear. For the case of the extension (figure 13*a*,*b*), the material system has a classical linear (quadratic in energy) behaviour up to λ≈0.4. Beyond this deformation, there is an onset of hardening as shown by the departure from the linearity in reaction (quadratic behaviour in energy). This sudden departure is particularly manifested in the case of *a*=0.01. In the case of bending (figure 13*c*,*d*), similar transition from a softer to stiffer material system is observable. However, in the case of shear (figure 13*e*,*f*), the material system retains a classical linear response (quadratic in energy) over the applied deformation range. Such a reversible phase transition in a material system from soft to stiff and vice versa in selected deformation modes could have many hitherto unprecedented applications. Particularly notable in this context is the transition in the contrast of extension stiffnesses to the shear stiffness of the material system. The strain energy under extension becomes an order of magnitude larger than that for shear, indicating a similar increase in the extensional stiffness over the shear stiffness. Similar phenomena are also observed with respect to the bending to shear behaviour although at a lesser magnitude. Pantographic structures are characterized at local level by the existence of floppy modes; more precisely by deformations (infinitesimals and large) corresponding to vanishing deformation energy. The manifold of floppy modes has a boundary; beyond some thresholds the pantographic sheet locally becomes a standard plate. This transition transforms a soft material into a stiff material. When many cells undergo this transition, then a global stiffening occurs (typically exponentially) and the specimen can be modelled as a first gradient material, for what concerns the in-plane gradient of displacements. The regions of soft second gradient behaviour and those of stiff first gradient ones are separated by boundary layers where a large gradient of bending and/or elongation occurs.

**Figure 14.**
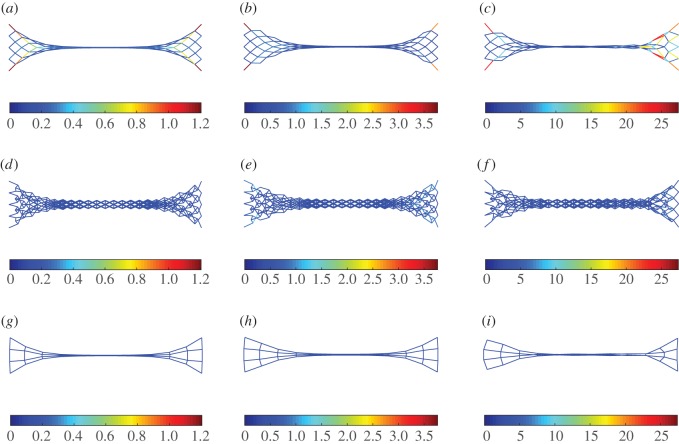
Extension test: deformation and strain-energy level (distinct for pantographic, king post and auxiliary rods) for λ=1 varying *a*=*a*_k_/*a*_p_. (*a*,*d*,*g*) *a*=0.01, (*b*,*e*,*h*) *a*=1, (*c*,*f*,*i*) *a*=100.

**Figure 15.**
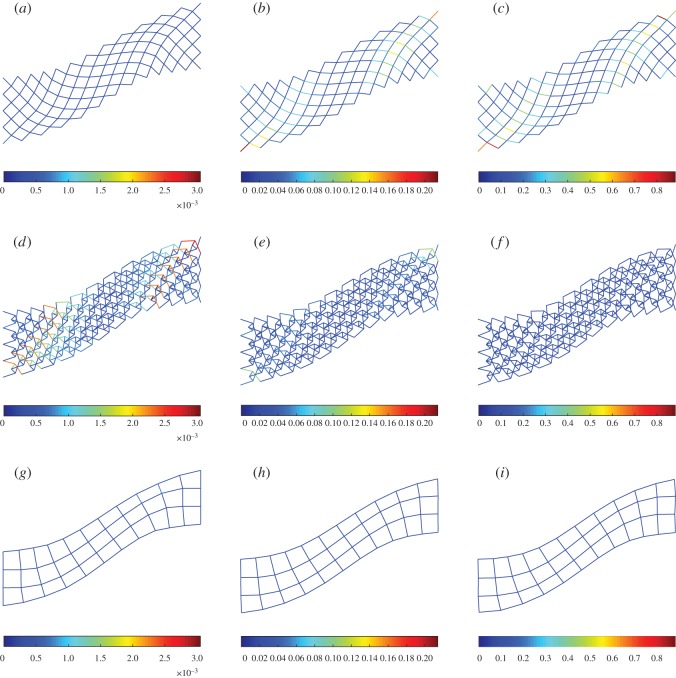
Shear test: deformation and strain-energy level (distinct for pantographic, king post and auxiliary rods) for λ=1 varying *a*=*a*_k_/*a*_p_. (*a*,*d*,*g*) *a*=0.01, (*b*,*e*,*h*) *a*=1, (*c*,*f*,*i*) *a*=100.

**Figure 16.**
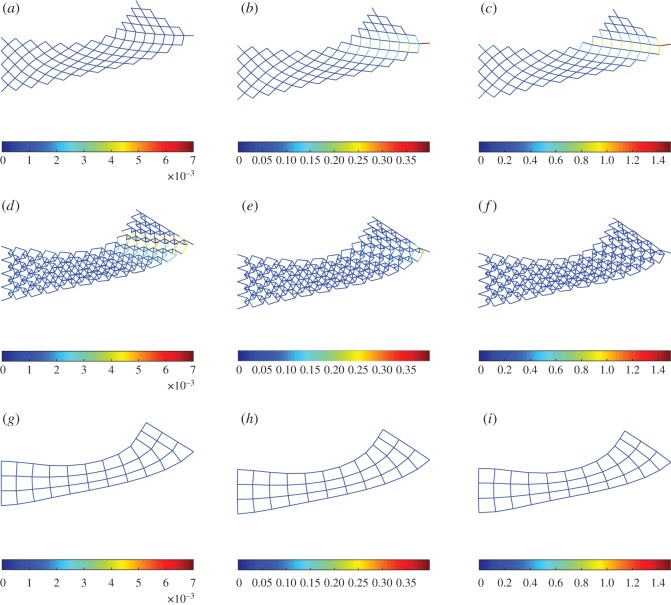
Bending test: deformation and strain-energy level (distinct for pantographic, king post and auxiliary rods) for λ=1 varying *a*=*a*_k_/*a*_p_. (*a*,*d*,*g*) *a*=0.01, (*b*,*e*,*h*) *a*=1, (*c*,*f*,*i*) *a*=100.

To explore further the macro-scale behaviour of the system from its building block scales, we show in figures 14–16 the deformed shapes at the end for extension, shear and bending tests, respectively, varying the stiffness parameter *a*=*a*_k_/*a*_p_. In all plots, the colour bar is used to represent the strain-energy level on the rods. For the sake of clarity pantographic, king post and auxiliary rods are plotted on separate layers. In the case of extension (figure 14), we observe that the auxiliary rods contribute negligible strain energy, while the pantographic rods contribute the most for the stiffness parameter *a*. In the case of bending and shear (figures 15 and 16), the pantographic and the king post rods contribute the bulk of the strain energy; however, the distribution depends upon the stiffness parameter *a*. For *a*<1, the king post rods have a larger contribution, while for *a*>1 the pantographic rods have the larger contribution. The contribution of auxiliary rods is negligible for the stiffness parameter *a*.

**Figure 17.**
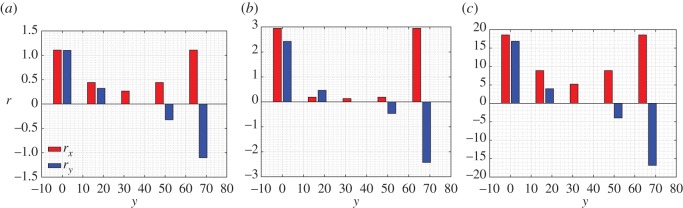
Extension test: structural reaction density *r* on the left-hand side (*x*=0) varying *a*=*a*_k_/*a*_p_ for λ=1. (*a*) *a*=0.01, (*b*) *a*=1 and (*c*) *a*=100.

Figure 17 reports, for the extension test, the structural reaction density both in the *x*_1_- and *x*_2_-direction varying the stiffness parameter *a*=*a*_k_/*a*_p_. The plots show the changes of the structural reaction distributions. Particularly interesting is the distribution of the horizontal component for the case *a*=1, which is very small compared with the values attained on the outermost.

## 4. A second gradient continuum model: numerical micro–macro identification based on presented discrete structure

In 1875, Alberto Castigliano, while investigating the mechanical behaviour of structures, formulated his celebrated theorems, which still supply a powerful investigation tool in linear and nonlinear elasticity (see [39–41]). Castigliano’s theorems are based on the validity of the principle of virtual work and are applicable for a large class of elastic systems. This class includes discrete Lagrangian systems, higher gradient continua and microstructured continua. If the set of external actions applicable to the considered elastic system is correctly individuated, then their expended work, in a quasi-static process, is exactly equal to the total deformation energy stored by the system in the same process. This statement, which is considered fundamental in classical thermodynamics, needs to be suitably formulated and exploited in the theory of elasticity. Castigliano astonished first Menabrea (see [42]) and then all mechanicians by getting out of it many fruitful and unexpected consequences in the theory of structures.

The applications conceived by Castigliano and his epigones concerned large length-scale systems of beams, like those needed for construction of bridges. However, the power of his method was not to be bound to such particular applications. One of the main tools used to obtain the results presented in this paper is again the idea subjacent to Castigliano’s theorems, as their scope surely includes also multi-scale structures whose various length scales can be varying, for instance, from 0.1 mm to 50 cm. The pantographic structures which we design here have to be built by standard devices used in additive printing. The elastic pivots interconnecting used beams may have diameters ranging from 0.1 mm onwards, the constituting beams may have sections whose diameter may range from 0.5 mm onwards and the distance between pivot may range from 0.5 to 2 mm, which is the same order of magnitude as the characteristic length of the periodicity cell in which the king post motif is incorporated. The constructed rectangular specimens may have a short side of a few centimetres and the long side of several decimetres. This is a multi-scale truss structure: therefore at every scale a specific model can (and must) be formulated.

At the smallest scale, all considered bodies may be modelled as first gradient continua (see [43]). At an intermediate meso-scale, the system can be modelled as a discrete system of extensional springs (as it is done in this paper in §2.1). Finally, at the macro length scale of the whole specimen a second gradient continuum model is more suitable. Each of these models must be elastic and for all of them Castigliano’s theorem applies, obviously also in the nonlinear regime. The identification process which equates the deformation energies of all these introduced elastic models for corresponding deformation states is the most efficient one, as it is strongly suggested by Castigliano’s theorems, and we have applied it here systematically. Also, after having started to apply Castigliano’s results to the design of metamaterials, we must continue to agree with Timoshenko (p. 292 of [44]): ‘Looking through all these applications, it is easy to see that little has been added to this branch of the theory of structures since Castigliano wrote his famous book’

Thus, at the macro-scale behaviour a novel continuum description is envisaged that can model the proposed metamaterial (e.g. [45–52] for a quick insight on this research line). To this end, we apply here a modified version of the second gradient model presented in [22]. Although the mathematical problems to be confronted in order to prove that such a continuum model is indeed the limit of the discrete one are rather difficult, we have observed numerically that indeed the continuum model captures rather efficiently the features of the discrete one also when the size of the characteristic cell is rather big. The mathematical methods to be used, for instance, to prove the *Γ*-convergence of the discrete model to a second gradient continuum one seem to be somehow similar to those exploited in [53]; however, as the problem discussed here involves two-dimensional pantographic structures in presence of extensible pantographic beams (those depicted in black in figure 2), the needed *a priori* estimates have not been found up to now. The present section therefore is further motivated by the need to develop understanding with numerical simulations that may direct the efforts needed to get a rigorous proof, which is, on the other hand, needed to understand the application range of continuum models.

We observe that:

(i) The geometry of a king post motif is not symmetric with respect to bending (or angular distortion). Consequently, under elongation, the equilibrium configuration is attained at a non-vanishing angle (figure 4) which manifests as curvature at the macro-level. This circumstance imposes certain properties on the macro-continuum model which is the homogenized limit of the considered microstructure. In future investigations we will introduce symmetric king post units in order to avoid that bending energy minima at non-vanishing angles and the elongation deformation energy remain quadratic in the considered pantographic unit.
(ii) The king post repetitive pantographic unit has been numerically studied and (figure 9) its deformation energy in flection depends on the bending angle *β* (previously called also angular distortion of the king post truss) via a third order (convex) polynomial in the neighbourhood of the reference configuration.

In view of the above observations, we adapted the conjectured second gradient continuum model as a limit model for considered pantographic structures in the case of moderate elongation energies and in the simultaneous presence of negligible localized bending deformation. The main features of the conjectured second gradient continuum model consist of an elongation energy which is quadratic and of a bending energy which is a suitable cubic polynomial in the referential curvature of pantographic fibres.^2^

To define the strain energy (see [22] for a deeper insight), we use the following strain parameters:

4.1 $$\begin{array}{rl} \varepsilon_{\alpha_{m}} & =∥\text{F}{\text{d}}_{\alpha_{m}}∥-1, \end{array}$$

4.2 $$\begin{array}{rl} \kappa_{\alpha_{m}} & =\sqrt{\frac{\nabla \text{F}|{\text{d}}_{\alpha_{m}}⊗{\text{d}}_{\alpha_{m}}\cdot \nabla \text{F}|{\text{d}}_{\alpha_{m}}⊗{\text{d}}_{\alpha_{m}}}{∥\text{F}{\text{d}}_{\alpha_{m}}{∥}^{2}}-{\left(\frac{\text{F}{\text{d}}_{\alpha_{m}}}{∥\text{F}{\text{d}}_{\alpha_{m}}∥}\cdot \frac{\nabla \text{F}|{\text{d}}_{\alpha_{m}}⊗{\text{d}}_{\alpha_{m}}}{∥\text{F}{\text{d}}_{\alpha_{m}}∥}\right)}^{2}} \end{array}$$

4.3 $$\begin{array}{rl} \;\text{and}\;\gamma & =\arcsin\left(\frac{\text{F}{\text{d}}_{\alpha_{1}}}{∥\text{F}{\text{d}}_{\alpha_{1}∥}}\cdot \frac{\text{F}{\text{d}}_{\alpha_{2}}}{∥\text{F}{\text{d}}_{\alpha_{2}}∥}\right), \end{array}$$

where **F**=∇*χ*, which is the gradient of the placement field *χ*, and having used the position:

4.4 $${\left(\nabla \text{F}|{\text{d}}_{\alpha_{m}}⊗{\text{d}}_{\alpha_{m}}\right)}^{\beta }=F_{\alpha_{m},\alpha_{m}}^{\beta },\;\text{(no sum over repeated }\alpha_{m}\text{ is intended)},$$

where the unit vectors **d**_*α*_*m*__ agree with the reference system axes.

With this notation, the strain energy can be written as

4.5 $$W(\chi )=\frac{1}{2}\int_{\Omega }\left(\sum_{\alpha_{m}}(A_{\alpha_{m}}\varepsilon_{\alpha_{m}}^{2}+b(\kappa_{\alpha_{m}},\varepsilon_{\alpha_{m}}))+\frac{1}{2}S\gamma^{2}\right)d\Omega ,$$

where a suitable function for the energy density related to the particular behaviour of the king post is used

4.6 $$b(\kappa_{\alpha_{m}},\varepsilon_{\alpha_{m}})=B_{1,\alpha_{m}}{\left(\kappa_{\alpha_{m}}-C_{1,\alpha_{m}}\varepsilon_{\alpha_{m}}\right)}^{3}+B_{2,\alpha_{m}}{\left(\kappa_{\alpha_{m}}-C_{2,\alpha_{m}}\varepsilon_{\alpha_{m}}\right)}^{2}$$

and the stiffness parameters of the continuum model (macro) *A*_*α*_*m*__, *B*_1,*α*_*m*__, *B*_2,*α*_*m*__, *C*_1,*α*_*m*__, *C*_2,*α*_*m*__ and S are related to the stiffness parameters of the discrete model (meso) *a*_p_, *a*_k_ and *a*_*x*_, i.e. to the stiffnesses of the pantographic, king post and auxiliary bars.

Analysing the different terms of equation (4.5), we can observe that the pantographic sheet behaves mechanically as a plane consisting of crossed rods of the Kirchhoff type arranged in two fibre families. Indeed, the measures of deformation appearing in the stored strain energy can be interpreted as the stretch of fibres (i.e. the elongation in the direction of each fibre), the fibre ‘curvature’ (i.e. the rate of change of the current tangent vector to the fibre with respect to arc length along the same fibre in the reference configuration) and the shear distortion (i.e. the change in the angle between fibres belonging to different families). We expect that in more general cases coupling bending/elongation effects will have to be taken into account by the introduction of an expression of bending deformation energy whose coefficients depend on elongation.

**Figure 18.**
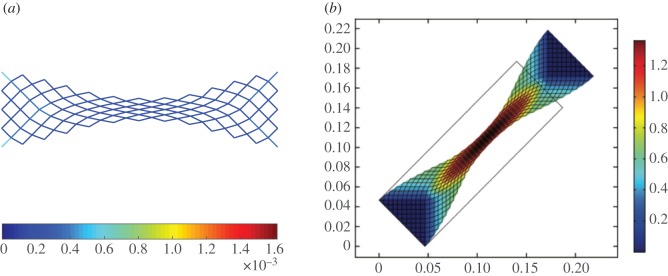
Extension test: comparison between deformation (λ=1) computed by the king post discrete model and the second gradient continuum model (*a*=*a*_k_/*a*_p_=0.01, $u_{\max}=0.45$). (*a*) king post discrete model and (*b*) second gradient continuum model.

**Figure 19.**
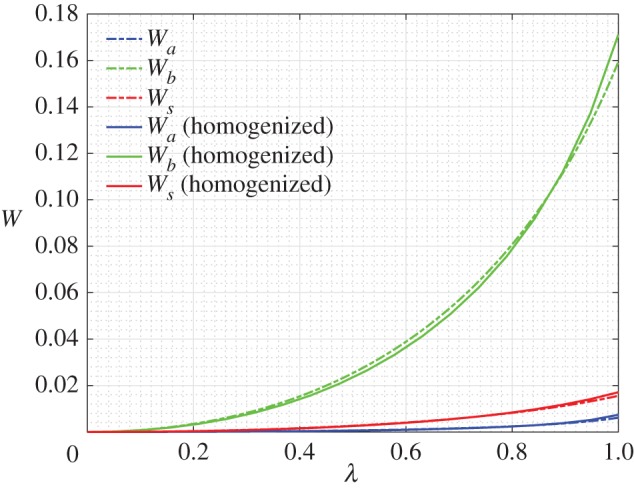
Extension test: strain energies varying the non-dimensional displacement parameter λ for the king post discrete model (dotted-dashed lines) and the second gradient continuum model (solid lines) grouped for pantographic (blue), king post (green) and auxiliary (red) bars (*a*=*a*_k_/*a*_p_=0.01, $u_{\max}=0.45$).

We have simulated the results of a simple extension test, such as reported in §3, but limiting in this case the maximum imposed displacement to $u_{\max}=0.45$. In figures 18 and 19, we compare the numerical simulations for the discrete system composed of repeated king post units and the numerical simulation for the corresponding second gradient continuum model whose constitutive parameters are found via a best fit procedure.

More precisely, in figure 18 there is a comparison between the deformation, corresponding to the maximum value of the imposed displacement (i.e. for the non-dimensional displacement parameter λ=1) obtained by using the king post discrete model (figure 18*a*) and the second gradient continuum model (figure 18*b*). Figure 18 also reports the energy density described by means of colours for the discrete model, whereas for the second gradient continuum model the colours indicate the strain parameter *γ* in which it is possible to see three characteristic zones with quasi-uniform deformation typical for pantographic structures. In figure 19 is shown the evolution of the strain energy *W* versus the non-dimensional displacement parameter λ. The strain energy is subdivided into three parts: the first one is related to the extension (in blue), the second one to the bending (in green) and the last to the shear (in red). Discrete and continuum model contribution can be identified by the kind of line used (continuous for the homogenized and dot-dashed for the discrete model). We stress that the strain energy for the discrete model is only related to the bar elongation, therefore we divided the strain energy into three parts according to our subdivision of the bars: pantographic bars give contributions on extensional strain energy, king post bars give contributions on bending strain energy and, finally, auxiliary bars give contributions on the shear strain energy.

Two further remarks are useful: (i) the parameters found for one precise imposed relative rigid translation of short sides very effectively can be used in all the other deformation problems such as those reported in §3, i.e. shear and bending tests; (ii) the macro-scale behaviour of presented metamaterial cannot be described without second gradient deformation energy. Indeed, the bending energy of pantographic elements, which in the presented microstructure is concentrated in the king post elements, is unavoidably related to directional second derivatives of displacement and, as shown in the present paper, the amount of deformation energy in such a bending mode is not negligible. On the other hand, when using only first gradient energies, some ill-posedness problems arise: indeed without second gradient energy any sharp jump of first gradient deformation can occur without energy and therefore possible solutions may arise, involving many different regions showing different deformation states. For more details see [56].

## 5. Concluding remarks

Metamaterial systems that exhibit controlled non-classical strain-energy functions can be constructed using a king post truss motif. We have demonstrated in this paper that the king post truss can be combined into pantographic sheet structures that have unprecedented strain-energy response. In the present paper, we (i) develop an effective computation tool for the study of the meso-model and, in particular, the king post motif forming pantographic sheets, (ii) determine the constitutive equations of the reduced-order model to be used at the length-scale characteristic of the specimen in terms of the mechanical properties of the king post motif considered, and (iii) prove that the consequent suitable second gradient model is effective in predicting at the macro-level the behaviour of pantographic sheets.

The presented simulations show that the strain-energy behaviour of the king post truss can be exploited in pantographic structures to obtain materials that exhibit reversible phase transition from soft to stiff and vice versa in selected deformation modes. This remarkable hierarchical material system is composed at the basic level of linear rods with classical quadratic strain energy. At the next level, these rods are combined to form the king post truss, which has a unique strain-energy landscape with respect to its degrees-of-freedom. The king post truss are then combined into pantographic units which individually exhibit certain abrupt changes in strain-energy behaviour. Finally, these pantographic units are combined by repetition to form the sheet structures wherein the cooperative behaviour of the units results in the observed non-classical behaviour. With the rapid advancement in additive manufacturing and 3D printing, such pantographic structures can indeed be realized as shown in [17,22,34]. As the resolution of these manufacturing techniques increase, it will be possible to create these structures at micro-scales with millions of basic building blocks. The simulations presented have shown that the proposed metamaterial has a wider elastic range, exhibits phase transition-type stiffening behaviour under particular tuneable loading condition, appears to be more damage resistant, is less susceptible to compression buckling and at the macro-scale displays behaviour characteristic of a second gradient continuum.

The proposed ‘metamaterial’ is characterized by many useful mechanical features and, thus, its technological applications can be related to different fields. In this regard, it is worth mentioning three possible areas of application: (i) in biomechanics, as prostheses in which the anisotropy due to the pantographic fibres disposition and the wide range of the elastic deformation could be required; (ii) in aeronautic, aerospace and naval engineering, due to the lightness of the resulting components as well as their high toughness and reliability; (iii) as impacting shields or bump devices with proper arrangement in sandwich composite structures for the possibility to store a considerable amount of elastic energy and then to dissipate it with proper dampers.

## Footnotes

1 More precisely, using the generally adopted terminology for structural trusses, we could distinguish the king post rod (in cyan) and the rafters (in red).

2 For an in-depth introduction on more refined beam models, the interested reader is referred to [54,55].

## Data accessibility

This article has no additional data.
